# Fatty Acid Composition of Gluten-Free Food (Bakery Products) for Celiac People

**DOI:** 10.3390/foods7060095

**Published:** 2018-06-20

**Authors:** Antonella Maggio, Santino Orecchio

**Affiliations:** Dipartimento di Scienze e Tecnologie Biologiche, Chimiche e Farmaceutiche, Università di Palermo, Viale delle Scienze, I-90128 Palermo, Italy; antonella.maggio@unipa.it

**Keywords:** gluten-free foods, fatty acids, GC–MS, celiac

## Abstract

The aim of this study (first analytical approach) was to obtain data concerning the fatty acid composition of gluten-free foods (bakery products) for celiac people. The study included 35 different products (snacks, biscuits, bakery products, pasta, flours, etc.) from several manufacturers. After extraction and esterification, the fatty acid composition was determined by Gaschromatography (GC–MS) Monounsaturated fatty acids (MUFAs) were found to be the major constituents (57%), followed by saturated fatty acids (SFAs) (30%), and polyunsaturated fatty acid (13%). Only 15 of the 35 gluten-free samples analyzed appeared to provide adequate energy intake, while, in 11 samples, saturated fatty acids were found to supply more energy than that recommended by the European Food Safety Authority EFSA. Moreover, data analyses showed that, although gluten-free commercial products are high added-value foods, industrial products in many cases contain palm and palm kernel oils, whereas the local producers generally use the finest raw materials, such as olive oil.

## 1. Introduction

Celiac disease is a persistent systemic autoimmune disorder caused by an enduring intolerance to gluten proteins in genetically predisposed people and is characterized by a wrong immune response of the T lymphocytes of the small intestine to gluten peptides [1]. This disease is characterized by chronic inflammation of the intestinal mucosa, atrophy of the intestinal villi, and several clinical manifestations.

Epidemiological research found that celiac disease is very common and that the prevalence of celiac disease is approximately 1% in the general population [2]. At present, the only available treatment is a lifelong strict gluten-free diet, which leads to the reinstatement of the atrophied intestinal villi.

The constant increase of the celiac populace leads to a higher demand of gluten-free food without wheat, rye, barley, or spelt wheat proteins (EU Regulation, 2014) [3]. Among these food types, gluten-free bakery products (bread, bread sticks, cookies, etc.) have significant advantages related to their high nutritional profile, ready-to-eat characteristic, variety of presentations and flavors. Common ingredients of gluten-free foods are starch and flour from corn, potato, tapioca, rice, etc. In several cases, these foods are integrated with vitamins and minerals [4,5]. Generally, gluten-free foods have high fat, sugar, and mineral contents to improve their flavor, consistency, and appearance [6]. Moreover, celiac people tend to offset the limitations of their diet by consuming food containing high levels of fat, sugar, and calories (snacks and biscuits, etc.). Saturni [6] affirms that celiac patients may show an excessive consumption of total fats and saturated fats. Other researchers confirmed that the alimentation of adolescent celiac people is hyperproteic and hyperlipidic, contains low amounts of carbohydrates, iron, calcium, fiber, etc. and, in several cases, is the principal cause of overweight [7,8,9]. It has been established that several gluten-free products contain trans-fatty acids [9] that may have a negative effect on health (leading to coronary heart disease, obesity, etc.).

Commonly, celiac people need assistance from a dietician that has no knowledge of the fatty acid composition of available gluten-free products; for this reason, it is necessary to know the composition of the foods consumed by individuals with celiac disease. While the macro- and microelements intake levels for celiac people are documented [10,11], there is no information on the fatty acids composition of gluten-free foods. Therefore, the aim of this study was to obtain information on the fatty acid content of several common gluten-free foods (bakery products) for celiac people, in particular, of several products sold in Italy (snacks, biscuits, bakery products, pasta, flours, etc.). In this research, the authors investigated 35 different products for celiac people from several manufacturers. After extraction and esterification, the fatty acid content was determined by GC–MS.

## 2. Materials and Methods

### 2.1. Quality Control and Quality Assurance

For each analytical batch, several blanks were run in order to demonstrate that the treatment used for cleaning the vessels and flasks was suitable to obtain the quality assurance required in this study. All the analyses of fatty acids in gluten-free food samples were repeated three times, and the relative standard deviation results ranged from 0.5 to 10%. The repeatability, calculated as the relative standard deviation (RSD%) of three independent measurements of a standard mix solution at 10 ng·mL^−1^, ranged from 0.3 to 8.0%. The repeatability of the whole method, calculated as the RSD%, for three independent analyses of different subsamples, ranged from 0.4 to 8.8%. All reported data were blank-corrected.

### 2.2. Samples

Thirty-five samples (Table 1) of gluten-free foods (pasta, biscuits, flours, etc.), produced and sold in Italy were collected from markets and pharmacies in the city of Palermo (Italy). The selected samples were representative of the Italian market. The data reported in Table 1 were obtained from the nutrition labels shown on the packs. The total fat content was verified by us on about 50% of the samples (see Section 2.3). Only the bread (sample *n*° 3) and breadstick (sample *n*° 7) were produced in local (Palermo and neighborhood) bakeries.

In order to obtain a representative sample, 50 g of each different sample was milled and homogenized by using a food processor (plastic-coated) and then sub-sampled for analysis. The samples were handled immediately or stored in a refrigerator at 4 °C until analysis for a period of less than 24 h.

### 2.3. Total Fats Quantification

The samples (5–10 g) were extracted by refluxing for 2–3 h with ethyl ether in a Soxhlet apparatus. The solvent was eliminated in a rotating evaporator, and the residue was dried at 70–80 °C for 30 min and weighted.

### 2.4. Fats Extraction and Preparation of Methyl Esters (FAMEs)

A portion of the homogenized samples (about 10 g) was extracted with a methanol/chloroform mixture according to Folch method [12]. The lipid extract was converted into fatty acid methyl esters (FAME) by treatment with 0.01 M sodium hydroxide in methanol at 60–65 °C, for 30 min at room temperature, followed by collection of the FAMEs dissolved in hexane (analytical grade from Sigma Aldrich, Milano, Italy).

### 2.5. GC–MS Analysis

The analysis of the standard (FAMEs) and samples was carried out using gas chromatography (Agilent Technologies 7890 B GC System) coupled with mass spectrometry (Agilent Technologies 7000 C GC/MS Quad.), using He 5.5 type as the carrier gas (flow 3 mL/min). An amount of 1 μL of each sample was injected. A capillary column (30 m × 0.25 mm i.d. × 0.25 μm film thickness) coated with a 5% phenyl-methylpolysiloxane stationary phase (DB-5 Agilent) was employed. The injector with a splitless system was set at 250 °C. The oven temperature was programmed at the beginning to 40 °C, then was increased to 250 °C with 2 °C/min increments (hold time 15 min), and finally reached 270 °C with 10 °C/min increments. The septum flow to the split vent was 3 mL/min, and the purge flow to the splint vent was 15 mL/min; the GC transfer line was set to 295 °C, and the source temperature to 200 °C. Using the above instrumental conditions, the different fatty acid methyl esters were clearly identifiable in the chromatograms; in detail, the compounds C18:1cis (R_t_ = 81.884 min), C18:1 trans (R_t_ = 82.183 min), C18:2 cis (R_t_ = 81.484 min), and C18:2 trans (R_t_ = 81.984 min) resulted well separated.

Ionization Energy was setted to 70 eV. Analyses were carried out in full-scan mode (with quantification based on the Total Ion Current (TIC)) as well as in SIM mode (**Selected Ion Monitoring**). All mass spectra were acquired over the *m*/*z* range 50–550, except during SIM. Three replicates were injected for each sample.

The data acquisition and processing were carried out using Mass Hunter Workstation Agilent Technologies Agilent software. The peaks of fatty acids were identified by comparison with those of the standards Supelco™ Component FAME Mix 37 and confirmed using the NIST mass spectral database. The confirmation of the structural and geometric isomers of fatty acids was carried out using Mix FAMEs C4:0–C24:1 and FAMEs Mix C20:1–C20:5.

**Figure 1:**
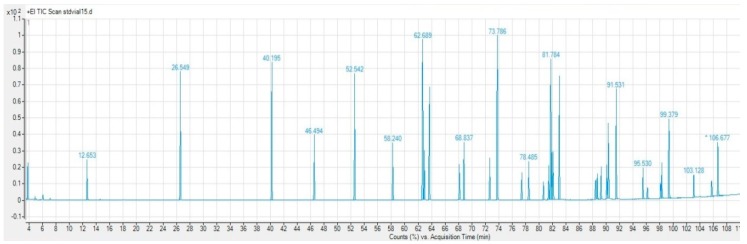
Chromatograms of a fatty acid methyl esters standard mix.

**Figure 2:**
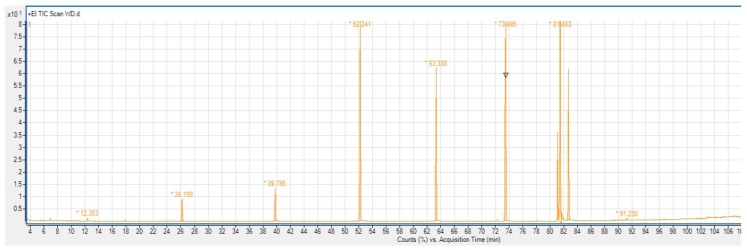
Chromatograms of the wafer sample.

Figure 1 and Figure 2 show the chromatograms of a standard fatty acid methyl esters mix and a wafer sample.

The quantification of the fatty acid methyl ester profiles was done considering the relative areas of the peaks, expressed as the relative percentage of the individual area of each peak relative to the total area of the peaks in the chromatogram.

## 3. Results

In the present study, 35 gluten-free food samples were analyzed from 2014 to 2017 to assess their nutritional characteristics with regard to the quantification of fatty acids. Overall, 37 fatty acids were detected (Table 2). The fatty acid compositions of the analyzed gluten-free foods are summarized in Table 2. The percentages of the single fatty acids indicated in the text (Table 2) are referred to the total fat content, while the total fat content (Table 1) refers to the food in the conditions in which it is consumed (g/100 g food). Data are shown only if the FA was present at >0.01% of total.

**Figure 3:**
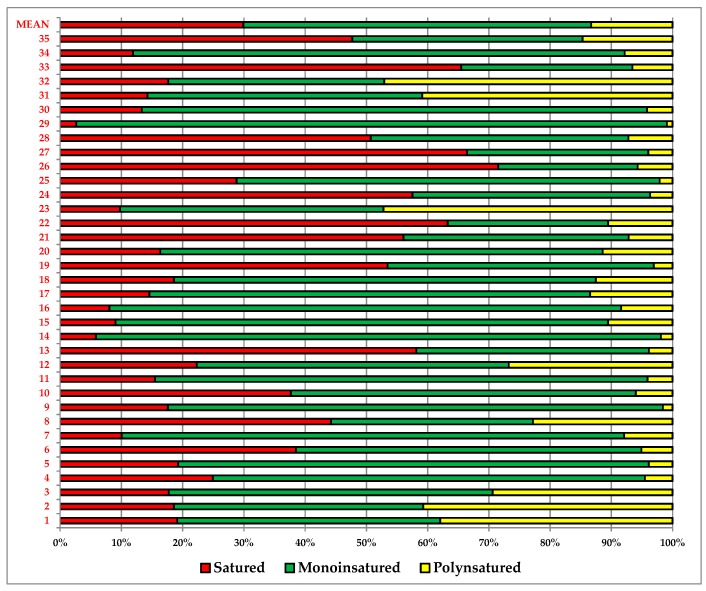
Saturated, mono-, and polyunsaturated fatty acids in the gluten-free samples.

The fatty acids identified and quantified were grouped into saturated fatty acids (SFA), monounsaturated fatty acids (MUFA), and polyunsaturated fatty acids (PUFA) (Figure 3).

In the analyzed samples, the mean percentage of the total lipids was 12.6%. The highest contents were found in the samples *n*° 26 (wafer biscuit) (25%) and *n*° 28 (muffin) (23%), while the lowest content was found in the sample *n*° 3 (flour mix for bread) (1.2%).

Saturated fatty acids were detected in all analyzed gluten-free food, and the percentages ranged from 2.8 to 72%. The highest contents were found in the samples *n*° 26 (wafer biscuit) (72%), *n*° 27 (Easter cake) (66%), and in the rice, corn, and red fruit flakes sample (*n*° 22) (63%), while the lowest (2.8%) content was found in in a biscuit sample (*n*° 29).

Palmitic acid (C16:0), the most prevalent SFA in the human diet, was measured as the major component, and high amounts of this fatty acid were detected in all samples. The highest content (51%) was found in the sample *n*° 13 (rosemary crackers), while the lowest content (1.3%) was in the sample *n*° 29 (biscuits *n*).

In the analyzed gluten-free samples, monounsaturated fatty acids ranged from 23 to 96%. The highest content was found in the sample *n*° 29 (biscuits *n*), while the lowest content was in the wafer biscuits (sample *n*° 26). Oleic acid was, quantitatively, the most important representative fatty acid in the studied samples; in fact, in several samples, monounsaturated fatty acids were constituted of oleic acid, which in the samples *n*° 26 (wafer) and *n*° 29 (biscuits n), respectively, ranged from 23% to 97%.

In the analyzed gluten-free food samples, other monounsaturated fatty acids, such as palmitoleic and eicosenoic acids, were present in lower amounts. In particular, they ranged from 0.11% (*n*° 17 apricot snacks) to 7.4% (*n*° 8 mini-breadsticks d) and from 0.08% (*n*° 32 nougat) to 2.1% (*n*° 8 mini-breadsticks) respectively.

The primary *n*-6 polyunsaturated fatty acids detected in our samples were linoleic, eicosatrienoic, and arachidonic acids. Linoleic acid ranged from 0.92 (*n*° 29 biscuits *n*) to 47.2% (*n*° 23 lemon biscuits).

Arachidonic acid was found only in the Easter cake (*n*° 27) and muffin (*n*° 28) samples at very low percentages (0.21 and 0.27%, respectively).

The only *n*-3 polyunsaturated fatty acid detected (at trace levels) in our samples was the docosahexaenoic acid. Traces of omega-3 fatty acid were found only in three samples (*n*° 24, 25, 27), while omega-6 fatty acid was contained in all samples, with percentages ranging from 1.2% to 48%.

Odd-chain fatty acids were identified in 29 gluten-free samples, with a mean value of 0.76%, and the percentages ranged from 0.02% (*n*° 16 corn and quinoa crackers) to 1.8% (*n*° 28 chocolate muffin). Trans-fatty acids were absent in all the analyzed gluten-free foods.

## 4. Discussion

An adequate quantity of fat in the diet is indispensable for health. In addition to its contribution to energy needs, the dietary fat must be sufficient to furnish the essential fatty acids and allow the absorption of fat-soluble dietary components such as some vitamins. The minimum quantities to ensure human health varies throughout a person’s life and among individuals. For example, an adequate intake of dietary fat is particularly important prior to and during pregnancy and lactation.

With regard to the total fat content in food, the National Institute of Health [13] proposes Recommended Daily Allowance (RDA) values only for children between 0 and 6 months and 6 and 12 months (31 and 30 g/day). This RDA is the average daily dietary intake level sufficient to meet the nutrient requirements of nearly all (97–98%) healthy individuals in a group. The Food and Agriculture Organization of the United Nations (FAO) [14] provides the recommended intakes of nutrients and the *safe levels of intake*, which apply to groups of persons and not to individuals (Table 3). In detail, they pertain to healthy, not diseased people. Based on the present knowledge, the reported values are designed to recommend intakes of fat that will maintain health, prevent deficiency diseases, and allow adequate fat stores in normal circumstances. The data for children report the amounts of fat that allow proper growth, and those for women of child-bearing age take into account their special needs, including those of pregnancy and lactation.

Considering the mean fat content in gluten-free foods analyzed in the present study (12.6%), it would be essential to consume from 182 to 365 g of gluten-free food to intake the requested amount suggested by the FAO (Table 3).

The total fat content of gluten-free flours is similar to that of traditional flours (flour 0, flour 00, rise flour, corn flour, etc.) (R_flour0_, R_flour00_, R_rice_, R_corn_,) [15,16] (Table 1 and Table 2).

**Figure 4:**
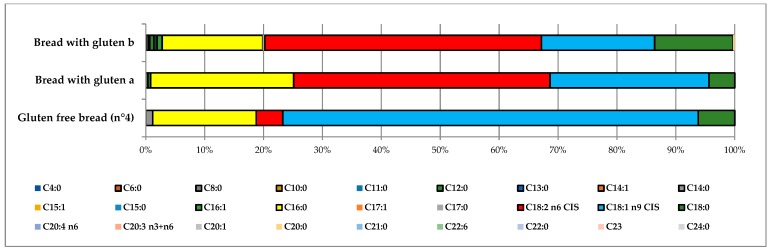
Comparison between the fatty acid distributions of gluten-free bread and of two types of bread containing gluten.

The handcrafted gluten-free bread produced in Palermo (sample *n*° 4) presented a total fat content (3.7%) similar to those reported in the literature (R_bread a_, R_bread b_) (2.1–3.5%) [15,16] but, compared to the industrially produced bread, contained a higher percentage of oleic acid (20% and 71%, respectively). On the other hand, sample *n*° 4 also contained a lower level of linoleic acid (4.5%) (Figure 4). Oleic acid is considered to be responsible for lowering the LDL–cholesterol levels in the blood. Moreover, oleic acid present in foods has preventive effects on several chronic diseases (cardiovascular diseases, cancer, or age-related cognitive decline). Like other fatty acids, the monounsaturated ones are almost completely absorbed from the intestine and are oxidized (for energy production), converted into other fatty acids, or incorporated into tissue lipids.

**Figure 5:**
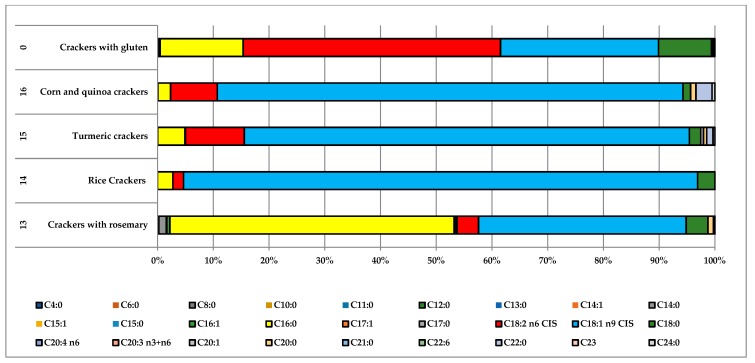
Comparison between the fatty acid distributions of the gluten-free crackers and gluten-containing crackers.

The sample *n*° 13 (rosemary crackers) differed from all the others gluten-free samples by its greater percentage (51%) of palmitic acid (Figure 5). Although it was produced by a multinational company, it can probably be assumed that palm or kernel oil was used.

Several relevant differences were highlighted in the composition of gluten-free wafers (sample *n*° 26) compared to traditional wafers. In detail, the total fat content (25%) was greater than in traditional wafers (Table 1) (R_w_) [15,16]. A similar situation was observed for the muffin sample (sample *n*° 28). Furthermore, in gluten-free wafers, dodecanoic and myristic acids were 21% and 13% respectively, an amount significantly higher than those of the other foods studied. These high percentages can be attributed to the use of palm kernel oil, a product largely available on the market and of low commercial value.

Arachidonic and eicosapentaenoic acids (the latter not found in the analyzed samples) can be further transformed to eicosanoids, a group of biologically active components, including prostaglandins, prostacyclins, and leukotrienes, which are very important in the regulation of blood pressure, renal function, blood coagulation, inflammatory and immunological reactions, and other functions [17].

Omega-3 fatty acids (α-linolenic acid) have anti-inflammatory properties and therefore may be useful in the management of inflammatory and autoimmune diseases [18]. The scientific results concerning the benefits deriving from the intake of omega-3-rich foods are discordant; a 2013 study states that in patients with multiple cardiovascular risk factors, a daily treatment with *n*-3 fatty acids did not reduce cardiovascular mortality and morbidity [19]. In the Western diet, the omega-6/omega 3 fatty acid ratio has been determined to range from 15/1 to 16.7/1 [19,20].

Simopoulos [20,21] indicated that people evolved on a diet with a ratio of omega-6/omega 3 essential fatty acids of ~1, whereas, in Western diets, the ratio is 15/1 to 16.7/1. A high omega-6/omega 3 ratio, as in our case and in the Western diets, could promote the pathogenesis of many diseases, including cardiovascular disease, cancer, osteoporosis, and inflammatory and autoimmune diseases, whereas increased levels of omega-3 polyunsaturated fatty acids exert suppressive effects.

The absence of trans-fatty acids in the gluten-free analyzed samples implies that bacterial transformation of unsaturated fatty acids (in the rumen of ruminant animals), industrial hydrogenation, deodorization of unsaturated vegetable oils, heating of oils at temperatures higher than 220 °C [17] were not carried out.

The EFSA Panel on Dietetic Products, Nutrition, and Allergies (NDA) [17] affirmed that precise quantities of fatty acids intake cannot be indicated; however, it is known that a fat intake lower than 35% (<35 E%, as energy production) produces a reduced energy intake and therefore weight reduction and/or prevention of weight increase. On the basis of practical considerations (e.g., current levels of intake, achievable dietary patterns), the EFSA Panel concluded that there are not sufficient available data to define a Lower Threshold Intake (LTI) or Tolerable Upper Intake Level (UL) for total fat intake, but only a Reference Intake range can be established. In European countries, no overt signs of deficiencies nor undesirable effects on blood lipids have been observed for intakes of total fats lower <20 E%, while intakes >35 E% may be compatible with both good health and normal body weight, depending on the dietary patterns and the level of physical activity. EFSA established for adults a lower bound of the Reference Intake range corresponding to 20 E% and an upper bound corresponding to 35 E%. The energy intakes from total fatty acids of the analyzed samples were calculated as reported in Equation (1).

E (%) = (L_total_·9 × 100)/CAL (1)

where L_total_ represents the fat content (g/100) that we determined in gluten-free food, and CAL (kcal) the energy supply of the food sample (obtained from the nutritional data provided on the package), respectively. The energy intake levels for celiac people were estimated for total lipids and for saturated fatty acids (Figure 4) based on the consumption of 100 g day^−1^ of gluten-free food.

**Figure 6:**
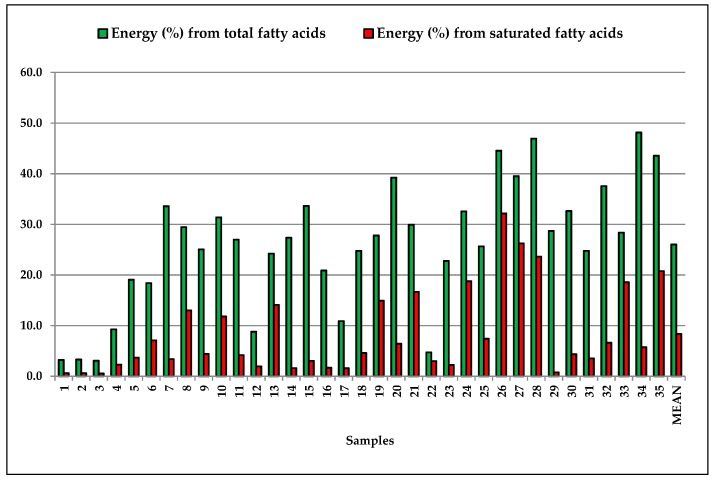
Energy (%) from lipids provided by 100 g of gluten-free samples.

In our case (Figure 6), the total lipids supply from 3.1 (*n*° 3, flour mix for bread) to 48% of energy (*n*° 34, biscuits), while the saturated fatty acids from 0.55 (*n*° 3, flour mix for bread) to 32% (*n*° 26 chocolate wafers). The results revealed that, on average, the monounsaturated fatty acids (MUFAs) constituted the majority (57%) of the fatty acids pool, followed by saturated fatty acids (SFAs) (30%) and polyunsaturated fatty acid (13%).

The saturated fatty acids of the analyzed samples supply from 0.55% (*n*° 3, mix flour) to 32% of energy (*n*° 26, chocolate wafers). On the basis of the EFSA [17] considerations, the saturated fatty acids should provide no more than 10% of energy. Only 15 of the 35 gluten-free samples analyzed provide an adequate energy intake, while, in 11 samples, the saturated fatty acids supply more energy than that recommended by EFSA.

Linoleic acid is essential in the diet because it cannot be synthesized by humans, and its deficiency results in unpleasant clinical symptoms, including scaly rashes and reduced growth; also, it is the precursor to arachidonic acid, which is the substrate for eicosanoid acid production in tissues. An Acceptable Macronutrient Distribution Range for linoleic acid is 0.6–1.2% of energy [17]. In our case, the energy obtained from the consumption of linoleic acid, present in gluten-free foods, varied from 0.92 (sample *n*° 29 biscuits) to 47% (sample *n*° 32 quinoa cake). The latter percentage corresponds to the highest linoleic acid intake from foods (no gluten-free) consumed by people in the United States and Canada [17]. High intakes of linoleic acid may constitute a protection against coronary heart diseases.

From a nutrition point of view, MUFAs have received growing attention because of their diverse effects on human health. Recent researches tend to indicate their beneficial effects, in particular, in reducing the risk of cardiovascular diseases and other inflammation-related diseases [22].

## 5. Conclusions

This paper is the first analytical approach to the study of 37 fatty acids in 35 different gluten-free foods produced in Italy for celiac people. The GC–MS technique was used to investigate the fatty acid composition. One of the advantages of the use of GC–MS for this characterization is its high sensitivity that improved the limits of quantification for analytes present at low levels in some samples. The data indicate a considerable variability between samples with respect to single fatty acid percentages, which could be due to the proportions of different ingredients contained in the analyzed foods.

From the analytical data, we can conclude that only about 40% of the samples analyzed in this study provide an adequate energy intake, and, in several samples, the saturated fatty acids provide more energy than that recommended by the EFSA.

Fortunately, from the nutritional point of view, celiac patients also consume foods of vegetable or animal nature (fresh) that compensate for any imbalances resulting from the use of bakery products of industrial origin.

It has emerged that local producers generally use the finest raw materials (olive oil, etc.) compared to the industries which, as has been pointed out, in many cases use palm and palm kernel oils, although gluten-free commercial products are high added-value foods, expensive, and intended for a particularly sensitive public.

## Figures and Tables

**Table 1:** Nutritional characteristics of the analyzed gluten-free samples and reference food (Rx).

*n*°	Sample	Energy (kcal/100 g)	Total Fat (g/100 g)	Saturated Fatty Acids (g/100 g)	Carbohydrates (g/100 g)	Proteins (g/100 g)	Salts (g/100 g)
Flours							
1	Rice flour	365	1.3	0.29	80.15	6.67	0.10
2	Red teff flour	380	1.4	0.27	-	-	-
3	Flour for bread	352	1.2	0.3	79.6	5.0	0.01
R_flour0_	Flour 0 with gluten	363	1.5	0.27	73.8	11.5	-
R_flour00_	Flour 00 with gluten	366	1.5	0.30	76.2	9.7	
R_rice_	Rice flour	366	1.4	0.39	80.1	5.9	
R_corn_	Corn flour	375	1.4	0.17	82.8	5.6	
Bread							
4	Handcrafted bread	360	3.7	0.90	-	-	-
R_breada_	Bread with gluten	271	3.5	0.86	50	8.8	
R_breadb_	White Bread with gluten	238	2.1	0.63	43.9	10.6	
Breadsticks							
5	Breadsticks a	425	9.0	1.8	75.0	2.4	1.9
6	Breadsticks b	416	8.5	3.2	79.9	4.0	2.7
7	Breadsticks c	462	17.2	2.54	73.57	0.99	1.6
8	Breadsticks d	446	14.6	2.9	75.0	2.4	1.9
9	Breadsticks with olive oil	431	12.0	3.5	79.0	1.30	2.12
10	Breadsticks with rosemary	460	16.0	5.13	76.32	2.04	2.80
11	Mini-breadsticks	427	12.8	1.7	74.9	1.4	1.2
Crackers							
12	Buckwheat crackers	369	3.6	0.6	72.0	9.1	2.0
13	Crackers with rosemary	446	12.0	7.1	79.0	3.3	1.3
14	Rice crackers	454	13.8	1.3	78.5	2.8	1.9
15	Turmeric crackers	455	17.0	1.8	66.0	7.6	2.1
R_crackers_	Crackers with gluten	421	8.9	2.0	74.3	9.5	-
16	Corn and quinoa crackers	422	9.8	0.9	79.5	1.8	1.7
Snacks							
17	Apricot snack	620	7.5	0.99	71.0	2.50	0.23
18	Crispy sheets	436	12.0	1.5	78.5	2.0	1.86
22	Rice and corn flakes	382	2.0	0.4	83.0	6.4	0.3
Biscuits							
19	Chocolate biscuits	453	14.0	7.70	77.0	4.0	0.55
20	Biscuits d	422	18.4	2.3	66.3	4.6	0.3
21	Biscuits f	451	15.0	7.8	72.0	5.4	0.4
23	Lemon biscuits	435	11.0	1.5	78.0	4.6	0.9
24	Seven-cereal biscuits	456	16.5	9.1	72.0	3.5	3.0
25	Biscuits o	456	13.0	4.6	83.0	2.0	0.57
26	Chocolate wafer	505	25.0	16.0	62.1	6.4	0.25
R_w_	Wafer with gluten	433	14.2	4.2	72.4	6.6	-
29	Biscuits n	439	14.0	4.2	69.4	7.0	0.7
30	Turmeric biscuits	441	16.0	1.8	78.0	2.3	0.33
31	Chocolate biscuits	425	11.7	2.7	73.6	5.1	0.8
33	Buckwheat biscuits	444	14.0	8.9	72.0	4.2	0.85
34	Biscuits c	372	19.9	2.5	52.8	4.4	0.07
35	Crunchy cereal biscuits	472	18.0	8.0	70.0	5.3	0.75
Muffins and cakes							
27	Easter cake	410	18.0	14.0	-	-	-
32	Quinoa cake	407	17.0	2.5	55.0	7.3	0.50
28	Chocolate muffin	441	23.0	8.6	52.0	3.9	0.5
R_m_	English muffin with gluten	223	2	0.29	44.8	8.7	-

Note: Breadsticks a, Breadsticks b, … Biscuit d, … etc. indicate the same type of food of different brans.

**Table 2:** Fatty acids composition (% of total fats) of gluten-free foods.

Fatty Acid	Samples																																			
	1	2	3	4	5	6	7	8	9	10	11	12	13	14	15	16	17	18	19	20	21	22	23	24	25	26	27	28	29	30	31	32	33	34	35	Mean
**C4:0**	0.01	0.01	0.01	0.01	0.01	0.01	0.01	0.01	0.01	0.01	0.01	0.01	0.01	0.01	0.01	0.01	0.01	0.01	0.22	0.01	0.01	0.01	0.01	1.29	0.21	0.01	0.08	0.01	0.01	0.01	0.01	0.01	0.4	0.01	0.01	0.071
**C6:0**	0.01	0.01	0.01	0.01	0.01	0.01	0.01	0.03	0.01	0.01	0.01	0.01	0.01	0.01	0.01	0.01	0.01	0.01	0.54	0.01	0.06	0.3	0.01	2.25	0.24	0.1	1.3	0.01	0.01	0.01	0.01	0.01	0.6	0.01	0.01	0.162
**C8:0**	0.01	0.01	0.01	0.01	0.01	0.04	0.01	0.04	0.01	0.01	0.01	0.01	0.01	0.01	0.01	0.01	0.01	0.01	0.42	0.01	0.8	1.84	0.01	3.06	0.23	1.7	0.87	0.01	0.01	0.01	0.01	0.01	0.94	0.01	0.01	0.291
**C10:0**	0.01	0.01	0.01	0.01	0.01	0.05	0.01	0.03	0.01	0.01	0.05	0.01	0.02	0.01	0.01	0.01	0.01	0.01	0.63	0.01	0.82	1.65	0.01	0.65	0.67	2.32	1.5	0.03	0.01	0.04	0.01	0.01	2.5	0.01	0.01	0.321
**C11:0**	0.01	0.01	0.01	0.01	0.01	0.01	0.01	0.01	0.01	0.01	0.01	0.01	0.01	0.01	0.01	0.01	0.01	0.01	0.06	0.01	0.04	0.01	0.01	0.14	0.01	0.02	0.03	0.01	0.01	0.01	0.01	0.01	0.01	0.01	0.01	0.017
**C12:0**	0.01	0.01	0.01	0.01	0.16	0.39	0.01	0.08	0.01	0.01	0.2	0.01	0.21	0.01	0.01	0.02	0.01	0.01	0.88	0.01	10.1	16.9	0.01	1.89	0.96	20.6	2.8	0.67	0.01	0.01	0.01	0.01	3.5	0.01	0.04	1.70
**C13:0**	0.01	0.01	0.01	0.01	0.01	0.01	0.01	0.01	0.01	0.01	0.01	0.01	0.01	0.01	0.01	0.01	0.01	0.01	0.1	0.01	0.01	0.01	0.01	0.45	0.04	0.04	0.09	0.01	0.01	0.01	0.01	0.01	0.11	0.01	0.01	0.03
**C14:1**	0.01	0.01	0.01	0.01	0.01	0.01	0.01	0.01	0.01	0.01	0.01	0.01	0.01	0.01	0.01	0.01	0.01	0.01	0.22	0.01	0.01	0.01	0.01	4.0	0.49	0.01	0.62	0.01	0.01	0.01	0.01	0.06	1.1	0.01	0.01	0.20
**C14:0**	0.18	0.01	17.7	1.2	0.01	1.3	0.01	0.32	0.1	0.01	0.79	0.05	1.3	0.0	0.03	0.05	0.09	0.17	3.3	0.2	4.6	10.6	0.05	8.11	3.15	12.6	9.3	0.26	0.01	0.16	0.06	0.12	11.0	0.04	0.64	2.50
**C15:1**	0.01	0.01	0.01	0.01	0.01	0.01	0.01	0.01	0.01	0.01	0.01	0.01	0.01	0.01	0.01	0.01	0.01	0.01	0.01	0.01	0.01	0.01	0.01	0.01	0.01	0.01	0.01	0.01	0.01	0.01	0.01	0.01	0.01	0.01	0.01	0.01
**C15:0**	0.01	0.01	0.01	0.01	0.01	0.1	0.01	0.01	0.01	0.01	0.05	0.03	0.11	0.01	0.01	0.0	0.01	0.02	0.64	0.05	0.17	0.01	0.01	0.7	0.6	0.08	0.76	0.16	0.01	0.02	0.01	0.03	1.44	0.01	0.03	0.15
**C16:1**	0.01	0.01	0.01	0.01	1.3	0.6	0.01	7.4	1.23	0.01	1.1	1.7	0.53	0.01	0.01	0.0	0.11	0.58	1.16	0.01	1.2	0.01	0.29	1.03	1.02	0.26	0.98	2.87	0.01	0.5	0.43	0.71	1.77	0.38	0.01	0.77
**C16:0**	15.4	13.6	0.01	17.6	11.5	33.3	4.5	22.8	11.9	34.3	7.4	18.6	51.1	2.8	4.9	2.2	9.7	10.9	29.4	10.1	33.3	24.0	6.1	28.0	16.0	22.0	34.6	33.4	1.3	4.1	8.9	12.1	28.9	4.8	41.0	17.4
**C17:1**	0.01	0.01	0.01	0.01	0.23	0.14	0.01	0.74	0.13	0.01	0.2	0.01	0.2	0.01	0.07	0.01	0.01	0.17	0.01	0.19	0.15	0.01	0.03	0.01	0.48	0.01	0.01	0.01	0.01	0.09	0.03	0.05	0.01	0.08	0.01	0.09
**C17:0**	0.01	0.01	0.01	0.01	0.15	0.19	0.01	0.51	0.12	0.01	0.12	0.01	0.31	0.01	0.03	0.02	0.01	0.13	0.32	0.08	0.33	0.01	0.03	0.19	0.28	0.2	0.31	1.12	0.02	0.06	0.06	0.06	0.7	0.04	0.05	0.158
**C18:3** ***n*6**	0.01	0.01	0.01	0.01	0.01	0.01	0.01	0.01	0.01	0.01	0.01	0.01	0.01	0.01	0.01	0.01	0.01	0.01	0.01	0.01	0.01	0.01	0.01	0.01	0.01	0.01	0.01	0.01	0.01	0.01	0.01	0.01	0.01	0.01	0.01	0.01
**C18:2 *n*6 cis**	38.0	40.7	29.4	4.5	3.9	5.1	7.9	22.8	1.6	6.0	4.1	26.8	3.9	1.9	10.6	8.4	13.5	12.5	3.1	11.4	7.1	10.5	47.2	3.2	2.1	5.75	3.58	6.9	0.92	4.2	40.9	47.0	6.55	7.84	14.7	13.3
**C18:1 *n*9 cis**	42.9	40.7	52.9	70.5	74.5	55.4	82.0	22.8	78.7	56.3	79.2	48.9	37.3	92.3	79.9	83.5	71.8	67.4	42.2	72.0	35.3	26.2	42.7	33.8	67.4	22.7	28.0	37.4	96.5	80.2	43.9	34.3	25.1	79.3	37.6	55.5
**C18:2 *n*6 tr**	0.01	0.01	0.01	0.01	0.01	0.01	0.01	0.01	0.01	0.01	0.01	0.01	0.01	0.01	0.01	0.01	0.01	0.01	0.01	0.01	0.01	0.01	0.01	0.01	0.01	0.01	0.01	0.01	0.01	0.01	0.01	0.01	0.01	0.01	0.01	0.01
**C18:1 *n*9 tr**	0.01	0.01	0.01	0.01	0.01	0.01	0.01	0.01	0.01	0.01	0.01	0.01	0.01	0.01	0.01	0.01	0.01	0.01	0.01	0.01	0.01	0.01	0.01	0.01	0.01	0.01	0.01	0.01	0.01	0.01	0.01	0.01	0.01	0.01	0.01	0.01
**C18:0**	3.0	4.8	0.01	6.2	5.9	2.3	4.6	16.7	4.2	3.3	1.4	3.2	3.9	3.1	2.0	1.4	4.0	5.4	16.7	4.9	4.2	8.0	2.8	9.2	4.9	12	14.2	8.7	0.23	1.4	2.5	3.7	12.2	2.6	5.8	5.41
**C20:4 *n*6**	0.01	0.01	0.01	0.01	0.01	0.01	0.01	0.01	0.01	0.01	0.01	0.01	0.01	0.01	0.01	0.01	0.01	0.01	0.01	0.01	0.01	0.01	0.01	0.01	0.01	0.01	0.21	0.27	0.01	0.01	0.01	0.01	0.01	0.01		0.02
**C20:5 *n*3**	0.01	0.01	0.01	0.01	0.01	0.01	0.01	0.01	0.01	0.01	0.01	0.01	0.01	0.01	0.01	0.01	0.01	0.01	0.01	0.01	0.01	0.01	0.01	0.01	0.01	0.01	0.01	0.01	0.01	0.01	0.01	0.01	0.01	0.01	0.01	0.01
**C20:3 *n*3+*n*6**	0.01	0.01	0.01	0.01	0.01	0.01	0.01	0.01	0.01	0.01	0.01	0.01	0.01	0.01	0.01	0.01	0.01	0.01	0.01	0.01	0.01	0.01	0.01	0.42	0.01	0.01	0.1	0.01	0.01	0.01	0.01	0.01	0.01	0.01	0.01	0.024
**C20:2 *n*6**	0.01	0.01	0.01	0.01	0.01	0.01	0.01	0.01	0.01	0.01	0.01	0.01	0.01	0.01	0.01	0.01	0.01	0.01	0.01	0.01	0.01	0.01	0.01	0.01	0.01	0.01	0.01	0.01	0.01	0.01	0.01	0.01	0.01	0.01	0.01	0.01
**C20:1**	0.01	0.01	0.01	0.01	0.97	0.47	0.01	2.09	0.7	0.01	0.01	0.36	0.01	0.01	0.49	0.01	0.01	0.73	0.01	0.01	0.01	0.01	0.01	0.01	0.01	0.01	0.01	1.6	0.01	1.7	0.49	0.08	0.01	0.85	0.01	0.307
**C20:0**	0.59	0.18	0.01	0.01	0.98	0.66	0.29	2.9	0.91	0.01	1.9	0.4	0.99	0.01	0.59	0.96	0.41	0.88	0.25	0.43	1.0	0.01	0.22	1.1	0.45	0.24	0.39	4.6	0.45	1.7	1.2	0.11	0.19	0.96	0.17	0.746
**C21:0**	0.01	0.01	0.01	0.01	0.05	0.01	0.01	0.01	0.01	0.01	0.01	0.01	0.01	0.01	0.01	0.01	0.01	0.01	0.01	0.01	0.01	0.01	0.01	0.06	0.01	0.01	0.01	0.01	0.01	0.01	0.01	0.01	0.01	0.01	0.01	0.013
**C22:6**	0.01	0.01	0.01	0.01	0.01	0.01	0.01	0.01	0.01	0.01	0.01	0.01	0.01	0.01	0.01	0.01	0.01	0.01	0.01	0.01	0.01	0.01	0.01	0.03	0.03	0.01	0.07	0.01	0.01	0.01	0.01	0.01	0.01	0.01	0.01	0.013
**C22:2**	0.01	0.01	0.01	0.01	0.01	0.01	0.01	0.01	0.01	0.01	0.01	0.01	0.01	0.01	0.01	0.01	0.01	0.01	0.01	0.01	0.01	0.01	0.01	0.01	0.01	0.01	0.01	0.01	0.01	0.01	0.01	0.01	0.01	0.01	0.01	0.01
**C22:1 *n*9**	0.01	0.01	0.01	0.01	0.01	0.01	0.01	0.01	0.01	0.01	0.01	0.01	0.01	0.01	0.01	0.01	0.01	0.01	0.01	0.01	0.01	0.01	0.01	0.01	0.01	0.01	0.01	0.01	0.01	0.01	0.01	0.01	0.01	0.01	0.01	0.01
**C22:0**	0.01	0.01	0.01	0.01	0.51	0.27	0.73	0.58	0.36	0.01	2.8	0.01	0.07	0.01	1.1	2.9	0.31	0.81	0.16	0.62	0.34	0.01	0.49	0.42	0.9	0.18	0.09	0.9	0.65	4.8	1.4	0.24	2.9	2.9	0.01	0.787
**C23**	0.01	0.01	0.01	0.01	0.01	0.01	0.01	0.05	0.12	0.01	0.07	0.01	0.01	0.01	0.01	0.01	0.01	0.01	0.01	0.01	0.01	0.01	0.02	0.04	0.01	0.01	0.02	0.7	0.01	0.07	0.01	0.16	0.01	0.01	0.01	0.043
**C24:0**	0.01	0.01	0.01	0.01	0.01	0.01	0.01	0.17	0.01	0.01	0.8	0.01	0.2	0.01	0.34	0.52	0.07	0.28	0.01	0.01	0.01	0.01	0.11	0.12	0.3	0.01	0.06	0.55	0.01	1.01	0.2	1.24	0.01	0.6	0.01	0.193
**C24:1**	0.01	0.01	0.01	0.01	0.01	0.01	0.01	0.01	0.01	0.01	0.01	0.01	0.01	0.01	0.01	0.01	0.01	0.01	0.01	0.01	0.01	0.01	0.01	0.01	0.01	0.01	0.01	0.01	0.01	0.01	0.01	0.01	0.01	0.01	0.01	0.01

**Table 3:** Average individual energy requirements and safe levels of fat intake.

Sex and Age Group	Weight (kg)	Energy (kcal)	Fat * (g)	Quantity of Gluten-Free Food Ensuring the Required Amount of Fat
Children				
6–12 months	8.5	950	-	-
1–3 years	11.5	1350	23–32	182
3–5 years	15.5	1600	27–62	214
5–7 years	19	1820	30–71	238
7–10 years	25	1900	32–74	254
Boys				
10–12 years	32.5	2120	35–82	278
12–14 years	41	2250	38–88	302
14–16 years	52.5	2650	44–103	349
16–18 years	61.5	2770	46–108	365
Girls				
10–12 years	33.5	1905	32–74	254
12–14 years	42	1955	33–76	262
14–16 years	49.5	2030	34–79	270
16–18 years	52.5	2060	34–80	270
Men				
18–60 years	63	2895	48–113	381
>60 years	63	2020	34–79	270
Women				
Not pregnancy or lactating	55	2210	37–86	294
Pregnancy	55	2410	40–94	317
Lactating	55	2710	45–105	357
>60 years	55	1835	31–71	246

* The fat requirements were calculated at the recommended range of 15 to 35 percent of the average energy requirements.
